# Postpartum acute stress disorder symptoms, social support, and quality of couple’s relationship associations with childbirth PTSD

**DOI:** 10.3389/fpsyt.2024.1310114

**Published:** 2024-06-10

**Authors:** Jonathan E. Handelzalts, Maor Kalfon-Hakhmigari, Adi Raichin, Yoav Peled

**Affiliations:** ^1^ School of Behavioral Sciences, Academic College of Tel-Aviv-Yaffo, Tel-Aviv, Israel; ^2^ Department of Psychiatry, University of Michigan, Ann Arbor, MI, United States; ^3^ The Helen Schneider Hospital for Women, Rabin Medical Center, Petach-Tikva, Israel; ^4^ Sackler Faculty of Medicine, Tel-Aviv University, Tel-Aviv, Israel

**Keywords:** social support, quality of couple’s relationship, PTSD, ASD, postpartum

## Abstract

**Background:**

We aimed to examine the hypothesized negative associations between childbirth post-traumatic stress disorder (PTSD) symptoms (using the two-factor model of birth-related and general symptoms), social support, and a couple’s relationship quality at 8–12 weeks postpartum. This analysis considered the longitudinal positive shared variance with acute stress disorder (ASD) symptoms measured shortly after birth, while accounting for obstetric and demographic variables.

**Methods:**

Participants included 246 mothers who gave birth at the maternity ward of a tertiary healthcare center. Self-report questionnaires were used 1–4 days postpartum (T1): Demographic information, the Birth Satisfaction Scale-Revised (BSS-R), and the National Stressful Events Survey Acute Stress Disorder Short Scale (NSESSS). At T2 (8–12 weeks postpartum), the Multidimensional Scale of Perceived Social Support (MSPSS), the Dyadic Adjustment Scale (DAS-7), and the City Birth Trauma Scale (BiTS).

**Results:**

In partial support of our hypotheses, three hierarchical regression analyses revealed a significant positive contribution of ASD symptoms to childbirth PTSD general symptoms (β = .33, *p* <.001) and the total score (β = .29, *p* <.001), but not to birth-related symptoms. Social support (β = -.21, *p* = .003) and the quality of the couple’s relationship (β=-.20, *p* = .003) showed negative associations with the BiTS general symptoms.

**Conclusion:**

Our study enhances understanding of the shared variance between childbirth ASD and PTSD, supporting the factor structure of general and birth-related symptoms as different aspects of childbirth PTSD and highlighting the negative association of social support and the quality of a couple’s relationship with PTSD general symptoms, suggesting potential avenues for targeted interventions.

## Introduction

Childbirth is usually considered a positive experience that can facilitate personal growth (1). However, in some cases, it can be associated with feelings of terror, fear, and helplessness (2), which in turn may predict adverse long-term consequences (3, 4). Approximately 30% of all women who give birth describe their birth experience as traumatic (5, 6). In this research, we aimed to study the association of women’s acute stress disorder (ASD) symptoms (as measured shortly after birth), social support, and couple’s relationship quality with childbirth posttraumatic stress disorder (PTSD) levels measured at 8–12 weeks postpartum.

PTSD is a disorder characterized by four main symptom clusters: re-experiencing, cognitive and behavioral avoidance, negative mood and cognition changes, and hyperarousal (7). Childbirth PTSD is thought to be not a generic form of PTSD that arises from the index event of childbirth but rather a potentially distinct subtype of PTSD (8), as childbirth is the only index event of trauma that is considered socially positive. Childbirth PTSD is also distinguished by its clinical characteristics and phenomenology, unique implications for the whole family, and the fact that the directly exposed survivors are always biologically women. It is further different in the sense of legitimacy and tendency to report, as well as the physiological processes involved (8).

Recent research regarding childbirth PTSD using a scale designed for that purpose [City BiTS; (9)] yielded a two-factor solution of birth-related symptoms and general symptoms (9–11). While the general symptoms factor presented a stronger correlation with measures of depression and anxiety, the birth-related symptoms factor presented stronger correlations with other measures of PTSD and aspects of childbirth (12–14).

A recent metadata reported that 4.7% of mothers met the criterion for PTSD following birth, while 12.3% of them exhibited significant levels of PTSD symptoms (15). This rate varies significantly depending on risk factors (15, 16). Commonly reported prepartum vulnerability factors included: belonging to a population at risk (this variable is defined differently across studies), depression in pregnancy, fear of childbirth, poor health or complications in pregnancy, a history of PTSD, and counseling for pregnancy or birth. Risk factors at birth included negative subjective birth experience, operative birth, lack of support, and dissociation. Postnatal vulnerability factors are depression, stress, and poor coping (15–18). As not all variables can be measured in all studies, in the current study, we used as covariates the type of birth and childbirth experience.

Acute stress disorder (ASD), which describes the initial response to a traumatic event, was introduced as a new diagnosis in the DSM’s fourth edition (19). Both ASD and PTSD are trauma- and stressor-related disorders. The diagnosis of PTSD comprises symptoms from each of the four different symptom clusters that must persist for at least 1 month, while for the diagnosis of ASD, nine symptoms from any of the five categories of intrusion, negative mood, dissociation, avoidance, and arousal must be present for up to 1 month (7). In general, ASD is considered a precursor of PTSD, though it is not considered a reliable predictor, as trauma-exposed people tend to follow one of four trajectories: (a) resilient, (b) worsening/delay onset, (c) recovery, and (d) chronically distressed (20, 21). Thus, while ASD can signal the development of PTSD, not everyone who experiences birth as a traumatic event and posttraumatic symptoms in the month after birth will necessarily develop PTSD (22). Nevertheless, ASD can interfere with early postnatal adjustment (23).

Although there is a lack of specific data regarding the transition from postpartum ASD to PTSD diagnosis, a recent study reported that following women from 8 weeks to 2 years postpartum for post-traumatic stress symptoms (PTSS), four latent classes differentiated by symptoms severity and type of symptoms were revealed: a High birth-related PTSS class (high probability to endorse all items, 4%), a Moderate birth-related PTSS class (moderate probabilities of endorsing intrusion symptoms, 16%), a Mild birth-related PTSS class (low probabilities of endorsing some of the items, 47%), as well as a No birth-related PTSS class (probabilities of above 0.9 for responding “not at all” to all items, 33%) (24). In addition, it was reported that ASD symptoms measured at 2–4 weeks postpartum were correlated with PTSD symptoms at 4 months for parents whose baby was admitted to a neonatal intensive care unit (25). Finally, ASD symptoms measured at 1-week postpartum predicted PTSD symptoms at 1-month postpartum (22).

In this study, apart from studying the shared variance of childbirth-related ASD and PTSD, we chose to focus on the context of women’s relationships as possible factors associated with childbirth PTSD. For that purpose, we examined the role of perceived social support and the quality of a couple’s relationship, as these are known resources that women may utilize after birth (26–28). We acknowledge that there are many other resources that may be associated with childbirth PTSD, such as maternal attachment style (29), though in this research we thought to focus on the perception of social support and relationships and not on the templates of relationships.

In general, PTSD and social support are known to predict each other bi-directionally (symmetrically and robustly), as PTSD symptoms may cause withdrawal from the environment, and the lack of social support may increase PTSD symptoms (30, 31). In the case of a traumatic birth experience, most studies reported the unidirectional connection of low social support increasing the probability of the development of postpartum PTSD and lowering the chances of recovery (26, 27).

The quality of a couple’s relationship plays a crucial role in displaying resilience during trauma, as romantic partners serve as essential resources (32). Childbirth PTSD has been found to be linked to reduced relationship satisfaction, though experiencing psychologically traumatic childbirth can lead to relationship strain but also strengthen relationships (33–35). In terms of partner support, women who perceived higher levels of their partner’s emotional support were less likely to develop symptoms of posttraumatic stress (36, 37).

As mentioned, there is some evidence for the negative association of social support and the quality of a couple’s relationship with childbirth PTSD. However, to our knowledge, no study tried to systematically measure both elements, considering postpartum ASD initial symptoms in a longitudinal design. Thus, we aimed to examine the associations between different aspects of childbirth PTSD symptoms (using the two-factor solution of birth-related and general symptoms) and support factors (social support and couple’s relationship quality) at 8–12 weeks postpartum, while considering the positive variance in ASD symptoms and the contribution of obstetric variables reported in the literature (birth type and birth experience). Specifically, the research hypothesis was that social support and couple’s relationship quality measured at 8–12 weeks postpartum would be negatively associated with PTSD total score, birth-related symptoms, and general symptoms at 8–12 weeks postpartum, beyond the positive association with ASD levels (measured a few days postpartum), even when controlling for birth type and birth experience (measured a few days postpartum).

## Methods

### Participants

The participants were women who gave birth in the maternity wards of Rabin Medical Center, a large tertiary healthcare center. Inclusion criteria were singleton pregnancy, being over 18, and Hebrew speaking. Initially, 964 women agreed to participate in the study, but only 806 had complete data at T1 (1–4 days postpartum). At T2, the number of participating women decreased to 246 (ages 20–47, *M*=32.68, *SD*=4.56). A comparison of the women who dropped out to those with complete data sets at both time points revealed no differences in ASD symptom levels at T1 (1–4 days postpartum). However, women who dropped out were younger (*t*(488.65)=3.01, *p* = .003) and had fewer years of education (*t*(458.44)=5.47, *p* < .001). In addition, a higher percentage of women who completed all time points identified as Jewish; χ² (1, *N*=867) =22.34, *p* < .001. The effect size was .16, *p* < .001, as measured by Cramer’s V. The sociodemographic and obstetric data of the study’s final sample (*N*=246) is presented in **Table 1**.

**Table 1:** Sociodemographic and obstetric data.

	*M*	*SD*
Age (years)	32.68	4.56
Education years	15.38	2.62
Marriage years	5.86	4.11
Birth experience	3.76	0.61
	*N*	*%*
Marital status		
Married or cohabiting	240	97.6
Not married	6	2.4
Religion		
Jewish	235	95.5
Not Jewish	11	4.5
Income level		
Below average	64	26.0
Average	88	35.8
Above average	91	37.0
Missing	3	1.2
First birth		
Yes	58	23.6
No	188	76.4
Planned delivery^a^		
Yes	190	77.2
No	28	11.4
Missing	28	11.4
Partner present at childbirth		
Yes	224	91.1
No	14	5.7
Missing	8	3.3
Newborn sex		
Men	112	45.5
Women	103	41.9
Missing	31	12.6
Administered epidural pain relief		
Yes	132	53.7
No	86	35.0
Missing	28	11.4
Induction of labor		
Yes	107	43.5
No	110	44.7
Missing	29	11.8
Premature birth		
Yes	11	4.5
No	205	83.3
Missing	30	12.2
Lacerations		
Yes	83	33.7
No	135	54.9
Missing	28	11.4

*N* = 246.

a Planned delivery: Yes [vaginal (n=156), elective cesarean section(n=34)], no [emergency cesarean section (n=21), assisted delivery (n=7)].

### Recruitment and procedure

The study was part of a more extensive longitudinal study aimed at understanding associations between factors linked with birth and postpartum mental health conducted between November 2020 and March 2022. Ethical approval for this study was obtained from Rabin Medical Center and the Academic College of Tel-Aviv Yaffo Institutional Review Boards. Data were collected in person for T1 (1–4 days postpartum). Research assistants approached all women in the maternity ward on a random day of the week. After giving informed consent, the participants completed a demographic questionnaire (including contact details such as email addresses and phone numbers), the National Stressful Events Survey Acute Stress Disorder Short Scale (NSESSS), and the Birth Satisfaction Scale—Revised (BSS-R). We chose to measure childbirth PTSD levels at T2 (8–12 weeks postpartum), as PTSD rates were found to be higher at 1–4 months postpartum and then decline (38) and large-scale childbirth PTSD studies like the INTERSECT measure within 6–12 weeks postpartum (14). At T2, participants completed the Dyadic Adjustment Scale (DAS-7), the Multidimensional Scale of Perceived Social Support (MSPSS), and the City Birth Trauma Scale (BiTS) using an online link sent by email. All participants who had not yet completed the online survey (T2) received an email reminder (3–5 days after the survey was sent) and a phone call (1–2 weeks after the survey was sent). Obstetric information was extracted from medical records. Questionnaires and data output were generated using Qualtrics^©^ 2019 (Qualtrics, Provo, UT; http://www.qualtrics.com).

### Measures

#### Sociodemographic and obstetric information

Sociodemographic questionnaires included age, education level, marital/cohabiting relationship, income level, and religious affiliation. Additionally, participants indicated if it was their first birth and if their partner was present at birth. Obstetric data were extracted from medical records, including the type of birth, epidural administration, labor induction, premature birth, and lacerations. Birth types were categorized into planned births, including vaginal births and elective cesarean births, and unplanned births, including emergency cesarean sections and vaginally assisted births (14).

#### Birth experience

Birth experience was measured using the Hebrew version of the Birth Satisfaction Scale-Revised [BSS-R; (39, 40)]. This 10-item 5-point Likert-type scale measures birth satisfaction using three sub-scales that measure the distinct but correlated domains of: (1) quality of care provision; (2) women’s personal attributes; and (3) stress experienced during birth. For this study, we used a total score ranging from 0 to 40, with higher scores indicating greater birth satisfaction and a better experience. Cronbach’s alpha in the original version is .79 (39). In the current study, internal consistency was good ω=.77.

#### Severity of acute stress symptoms

The severity of acute stress symptoms was measured using the National Stressful Events Survey Acute Stress Disorder Short Scale [NSESSS-PTSD; (41)]. This is a 7-item, 5-point Likert scale questionnaire assessing the severity of symptoms consistent with acute stress disorder in adults. Participants were asked to rate the severity of their symptoms over the past 7 days. The score ranges from 0 to 28, with a higher score indicating a higher severity of ASD symptoms. Cronbach’s alpha of the NSESSS was found to be.91 (42). In the current study, internal consistency was good ω=.83.

#### Perceived social support

Perceived social support was measured using the Hebrew version of the Multidimensional Scale of Perceived Social Support [MSPSS; (43, 44)]. This is a 12-item, 7-point Likert scale, ranging from 12 to 84, designed to assess the individual’s present perception of social support over three dimensions: family, friends, and significant others. Higher scores indicate a better perceived experience. The Cronbach’s alpha for the MSPSS was .88 (43). In the current study, internal consistency was good: ω = .93 for the total score, ω = .89 for family, ω = .95 for friends, and ω = .87 for significant others.

#### Quality of a couple’s relationship

The quality of the couple’s relationship was measured using the Hebrew version of the Dyadic Adjustment Scale (DAS-7) (45, 46). A 7-item Likert scale, ranging from 7 to 43, assesses the value of agreement, frequency of activity together, and happiness with the current relationship, with higher sum scores indicating higher relationship quality. The Cronbach’s alpha for the Hebrew version was found to be .79 (46). In the current study, internal consistency was good, ω = .83.

#### Childbirth PTSD symptoms

Childbirth PTSD symptoms were measured using a validated Hebrew version of the City Birth Trauma Scale [BiTS; (9, 47)]. A 31-item questionnaire was developed to measure birth-related PTSD according to DSM-5 criteria (7): Twenty-three items assess the frequency of symptoms over the last week, scored on a scale ranging from 0 = *not at all* to 3 = *5 or more times*, and cover four symptom clusters of the DSM-5: ‘re-experiencing’, ‘avoidance’, ‘negative mood and cognitions’, and ‘hyperarousal’. For this analysis, we used the BiTS two symptom factors (9, 12, 47): birth-related symptoms (BRS; covering symptoms of intrusions, avoidance, and two items from negative cognitions and mood specifically related to birth) (9 items; range 0–27) and general symptoms (GS; covering other items from negative cognitions and mood and hyperarousal) (11 items; range 0–33). Cronbach’s α of .90 was for the Hebrew version (47), and in the current study, the internal consistency was found to be good for the BRS (ω = .87), the GS (ω = .89), and the total score (ω = .87).

### Statistical analysis

To determine the appropriate sample size for our study, we conducted a power analysis using the G*Power program. Based on an expected medium effect size, a power of 0.95, and an alpha probability error of 5% for five predictors (including covariates), we calculated that a minimum of 138 participants would be required. Two hundred and nine participants had complete data sets at both time points. We added another 37 with less than 20% missing values using the relevant mean value (48). Specifically, for item number 7 of the DAS-7 questionnaire, which was measured on a scale of 1–7, unlike the other items that were measured on a scale of 1–6, any missing values were substituted using linear regression based on the responses from the other six DAS-7 questions. Data were described as mean and *SD* or as counts and percentages. Correlations between the study variables were assessed using the Pearson correlation coefficient. The outcome variables, BiTS total score, birth-related symptoms, and general symptoms, were modeled using hierarchical linear regression. The predictors were ASD symptoms, social support, and the couple’s relationship quality. Birth experience and the birth type (planned or unplanned birth) were entered for the analysis due to the studies reporting them as possible PTSD antecedents (see introduction). In our study, birth experience was statistically associated with BiTS total score, birth-related symptoms, and general symptoms. Birth type had a statistically significant association with BiTS total score and birth-related symptoms. The analysis did not include other covariates, as no other demographic/obstetric variable measured in our study had a significant statistical association with the dependent variables. ASD symptoms, birth experience, and birth type were entered in the first step. Social support and the couple’s relationship quality were both entered in the second, as they were both measured at the same timepoint (T2, 8–12 weeks postpartum). Descriptives and correlations between social support subscales are presented in **Table 2**. As we were interested in social support in general, due to the high correlations between the subscales, we used only the social support total scale. Results were reported as estimated beta (β) standardized coefficients, unstandardized coefficients (*B*), standard error (*SE*), and associated *p*-value. The adjusted explained variance (*R* ^2^) and associate *F* value were reported in each step as well. A significance level of *p* <.05 was used for statistical significance. However, as three hierarchical regression analyses were conducted on the same data, a Bonferroni correction of.016 was used to control for familywise errors. Data were analyzed using the statistical software package SPSS 28.0 (SPSS Inc., Chicago, IL).

**Table 2:** Means, standard deviations, and correlations of outcome variables.

Variables	*M*	*SD*	1	2	3	4	5	6	7	8	9
1. NSESSS-PTSD	2.99	3.90	–	-.10	-.26***	-.21***	-.23***	-.23***	.42***	.28***	.42***
2. DAS-7	33.09	5.51		–	.46***	.41***	.33***	.50***	-.36***	.01	-.23***
3. MSPSS	6.03	1.04			–	.84***	.89***	.82***	-.40***	-.14*	-.34***
4. MSPSS - family	6.10	1.15				–	.57***	.63***	-.37***	-.15*	-.33***
5. MSPSS - friends	5.54	1.62					–	.59***	-.33***	-.13*	-.28***
6. MSPSS - significant others	6.47	0.88						–	-.35***	-.07	-.27***
7. BiTS- GS	5.24	5.71							–	.37***	.86***
8. BiTS- BRS	2.47	4.63								–	.78***
9. BiTS- total score	7.88	8.64									–

*N*= 246. * *p* <.05, ** *p* <.016, ***p <.001. NSESSS-PTSD, The National Stressful Events Survey Acute Stress Disorder Short Scale for assessing acute stress symptoms severity; DAS-7, The Dyadic Adjustment Scale for couple’s relationship quality; MSPSS, The Multidimensional Scale of Perceived Social Support; BiTS, The City Birth Trauma Scale for assessing childbirth PTSD symptoms.

## Results

**Table 1** presents the sample demographic and obstetric data. The associations between demographic and obstetric variables and the criterion variables of our models—BiTS birth-related symptoms, general symptoms, and the total score—were calculated. The results showed no significant correlations or differences between the demographic variables and the criterion variables. However, birth experience and the birth type [planned (vaginal birth and elective cesarean section) or unplanned (vaginally assisted birth and emergency cesarean section)] that were chosen as covariates following the literature review and entered into the analysis also had statistically significant associations with the dependent variables, as reported in the statistical analysis section. Descriptive statistics of the outcome measures and the correlations among them are presented in **Table 2**.

To test the hypotheses and examine whether social support and the quality of a couple’s relationship measured at 8–12 weeks postpartum will explain PTSD symptoms at 8–12 weeks postpartum beyond ASD levels measured a few days postpartum, we conducted three hierarchical regressions with BiTS general symptoms, BiTS birth-related symptoms, and BiTS total score as the criterion. The results are presented in **Tables 3**–**5**, respectively. All three models were significant at both steps of each regression. ASD symptoms significantly contributed positively to the variance of general symptoms and the total score in both models’ steps but did not contribute significantly (Bonferroni correction) to birth-related symptoms. Social support (β = -.21, *p* = .003) and the quality of the couple’s relationship (β= -.20, *p* = .003) had significant negative contributions to explaining postpartum BiTS general symptoms but did not have significant contributions regarding BiTS birth-related symptoms or the total score. Birth experience (β = .28, *p* <.001) and birth type (planed vs. unplanned) (β = .21, *p* = .007) had significant contributions to explaining postpartum BiTS birth-related symptoms. The results partially supported our hypotheses, as social support and the quality of the couple’s relationship contributed differently to BiTS birth-related symptoms, general symptoms, and the total score.

**Table 3:** Hierarchical regression analysis with a postpartum PTSD general symptoms as a criterion.

Predictors	β	*B*	*SE (B)*	
Step 1- T1 1–4 days postpartum				
(constant)	–	9.93	3.17	*R* ^2^ = .22
Unplanned delivery	-.05	-0.91	1.18	
NSESSS-PTSD	.40***	.590	0.10	*F*(3, 204) = 19.0***
BSS-R	-.15*	-1.43	0.67	
Step 2- T2 8–12 weeks postpartum				
(constant)	–	22.07	3.58	
Unplanned delivery	-.01	-0.02	1.10	
NSESSS-PTSD	.33***	.490	.100	*R* ^2^ = .12
BSS-R	-.11	-1.04	.630	
MSPSS	-.21**	-1.19	.400	∆*R* ^2^ = .34
DAS-7	-.20**	-0.22	.070	*F*(5, 202) = 20.40***

*n*=208. *p <.05, **p <.016, ***p <.001.

The lower n is due to missing data regarding demographic information. PTSD, post-traumatic stress disorder; NSESSS-PTSD, The National Stressful Events Survey Acute Stress Disorder Short Scale for assessing acute stress symptoms severity; BSS-R, The Birth Satisfaction Scale—revised; MSPSS, The Multidimensional Scale of perceived social support; DAS-7, The Dyadic Adjustment Scale for a couple’s relationship quality.

**Table 4:** Hierarchical regression analysis with a postpartum PTSD birth-related symptoms as a criterion.

Predictors	β	*B*	*SE (B)*	
Step 1- T1 1–4 days postpartum				
(constant)	–	6.87	2.37	*R* ^2^ = .22
Unplanned delivery	.18**	2.44	0.88	*F*(3, 204) = 18.74***
NSESSS-PTSD	.16*	0.18	0.08	
BSS-R	-.29***	-2.09	0.50	
Step 2- T2 8–12 weeks postpartum				
(constant)	–	7.21	2.90	
Unplanned delivery	.18**	2.43	0.89	
NSESSS-PTSD	.15*	0.17	0.08	∆*R* ^2^ <.01
BSS-R	-.28***	-2.04	0.51	
MSPSS	-.04	-.16	0.32	*R* ^2^ = .22
DAS-7	.02	0.02	0.06	*F*(5, 202) = 11.20***

*n*=208. *p <.05, **p <.016, ***p <.001.

The lower n is due to missing data regarding demographic information. PTSD, post-traumatic stress disorder; NSESSS-PTSD, The National Stressful Events Survey Acute Stress Disorder Short Scale for assessing acute stress symptoms severity; BSS-R, The Birth Satisfaction Scale—revised; MSPSS, The Multidimensional Scale of perceived social support; DAS-7, The Dyadic Adjustment Scale for a couple’s relationship quality.

**Table 5:** Hierarchical regression analysis with a postpartum PTSD total score as a criterion.

Predictors	β	*B*	*SE (B)*	
Step 1- T1 1–4 days postpartum				
(constant)	–	16.70	4.63	*R* ^2^ = .26
Unplanned delivery	.07	1.84	1.72	*F*(3, 204) = 24.02***
NSESSS-PTSD	.34***	0.75	.150	
BSS-R	-.24***	-3.52	0.98	
Step 2- T2 8–12 weeks postpartum				
(constant)	–	29.0	5.46	
Unplanned delivery	.10	2.71	1.68	
NSESSS-PTSD	.29***	0.65	0.14	∆*R* ^2^ = 05
BSS-R	-.21**	-3.10	0.96	
MSPSS	-.15*	-1.32	0.60	*R* ^2^ = .31
DAS-7	-.12	-0.20	0.11	*F*(5, 202) = 18.56***

*n*=208. *p <.05, **p <.016, ***p <.001.

The lower n is due to missing data regarding demographic information. PTSD, post-traumatic stress disorder; NSESSS-PTSD, The National Stressful Events Survey Acute Stress Disorder Short Scale for assessing acute stress symptoms severity; BSS-R, The Birth Satisfaction Scale—revised; MSPSS, The Multidimensional Scale of perceived social support; DAS-7, The Dyadic Adjustment Scale for a couple’s relationship quality.

## Discussion

This study investigated the associations between aspects of childbirth PTSD and the mother’s perceived support systems through the perspective of the childbirth PTSD total score and the two-factor model: birth-related symptoms and general symptoms [e.g., (9)]. Both factors and the total score were positively associated with ASD symptom levels. However, in partial support of our hypotheses, while considering the presence of the covariates of birth experience and birth type, we found that ASD significantly contributed to general symptoms and the total score but not to birth-related symptoms. In addition, in partial support of our hypotheses, both social support and perceived quality of couples’ relationships had significant negative associations with childbirth PTSD general symptoms; contrary to our hypotheses, neither the social support nor perceived quality of couple’s relationship levels were associated with birth-related symptoms and the total score in models considering ASD symptoms as well as birth experience and birth type.

We measured postpartum trauma-related symptoms at two time points, measuring symptoms of two similar but different disorders: ASD and PTSD. While both are characterized by a similar set of symptoms, they differ in the timing of diagnosis (7). Although, as mentioned above, ASD may be a precursor but not a reliable predictor for PTSD [e.g., (20)], our results indicate that there is an association between ASD and specific PTSD symptoms levels (general symptoms), though other PTSD symptoms (birth-related) were not associated with ASD in the final model. ASD, along with the other variables, accounted for approximately 31% of the variance in the total BiTS score, 22% in the birth-related symptoms score, and 34% in the general symptoms score. ASD contributed significantly, with ASD β values of.29 to the BiTS total score,.33 to the BiTS general symptoms, and only.15 to the BiTS birth-related symptoms (which was only close to significance in our model).

Thus, although high postpartum ASD symptom levels are not considered predictors for PTSD symptoms, they may serve as a possible warning sign for specific (e.g., general PTSD symptoms) long-term consequences for vulnerable patients who may develop persistent trauma (49), though this may depend on specific individuals’s symptoms trajectories (24). We suggest that the inconsistent results for the relationship between postpartum ASD and PTSD symptoms may be associated with the fact that ASD symptom levels were specifically related to childbirth-related PTSD symptoms, though unrelated to the more specific birth-related symptoms. Currently, there is insufficient literature investigating the possible role of ASD in predicting traumatic symptoms in the later stages of the postpartum period (22), and our study adds to the understanding of the shared variance between childbirth ASD and PTSD symptoms by considering the different childbirth PTSD factors (22, 25).

It was previously established that the symptoms’ scores measured using the BiTS questionnaire could be clustered into two distinct factors: birth-related symptoms and general symptoms, while no support was found for the DSM’s four-symptom cluster solution (9–14, 47). Specifically, intrusions and avoidance symptoms were found to load on the birth-related symptoms factor, while hyperarousal symptoms were found to load on the general symptoms factor. Symptoms from the negative cognition and mood cluster, which was added to the DSM-5, split across the two factors [e.g., (12, 13, 47)]. Our study provides support for the differentiation between birth-related and general symptoms on various levels. While birth-related symptoms were found to be associated with childbirth experience and birth type, they were not associated with previous ASD levels, social support, or the quality of a couple’s relationship. Childbirth PTSD general symptoms, on the other hand, were associated with previous ASD levels as well as with social support and the quality of the couple’s relationship, but not with childbirth experience or birth type.

We report, in similarity to previous research, that birth-related symptoms were found to be more sensitive to whether the birth went as planned (either elective cesarean section or vaginal birth) or not (emergency cesarean section or instrumental vaginal birth), and unplanned birth was associated with higher birth-related symptoms (12–14). Although, to the best of our knowledge, no studies measured specific birth-related PTSD symptoms or childbirth experience, the results adhere to the studies reporting childbirth experience as an antecedent to childbirth PTSD total scores (17, 50). Birth-related symptoms that are compromised by avoidance and intrusion symptoms, as well as some of the negative emotions and cognition symptoms (items that are specifically related to the birth, e.g., “feeling strong negative emotions about the birth”), were not associated in our study with the perceived quality of a couple’s relationship or social support levels. We suggest that while birth-related symptoms are less sensitive to perceived support resources and initial levels of ASD symptoms, they may be more sensitive to specific variables associated with the birth process, though more research is needed.

On the other hand, childbirth PTSD general symptoms that are compromised by hyperarousal symptoms and some of the negative emotions and cognitions (more general items, e.g., “feeling detached from other people”) were associated in our study with both perceived social support and the quality of the couple’s relationship, even when considering baseline ASD levels. Thus, stressing the important role of perceived social resources vis-à-vis these symptoms. Social support and the quality of a couple’s relationship were found to be associated with childbirth PTSD as measured by a single-factor scale [e.g., (26, 34, 35)]. However, our study results point to the possibility that these perceived social resources may be associated with the more long-term consequences of childbirth PTSD general symptoms, but not with the more specific birth-related symptom levels.

### Limitations and future studies

This study is not without limitations. First, all the participants were sampled in a specific maternity ward and were mainly married Jewish women whose partner was present at childbirth, therefore limiting generalizability. Further, we grouped birth types as planned (vaginal and elective cesarean sections) vs. unplanned (vaginally assisted and emergency cesarean sections). Future studies should include more diverse samples concerning race, gender (including fathers), family structure, etc., with a higher number of participants in order to allow for a closer look at the effect of specific birth types. Second, we had a low retention level (only 27% of the initial sample collected at Time 1), and women dropping out of the survey tended to be non-Jewish, although they were not different in their initial ASD levels. Nevertheless, this may limit generalizability, in addition to the fact that women who joined the research in the beginning may have been different than women who chose not to participate. A third limitation was the use of self-report measures. That said, the self-report scales used for this research are valid, reliable, and widely used in the literature. A fourth limitation concerns the wide array of potential factors that may be associated with childbirth PTSD that were not measured in this study. Although we controlled for the two major possible covariates mentioned in the literature, many other factors, such as communication with staff, previous traumatic experiences, or other factors, may influence childbirth PTSD and should be considered in future research. In addition, as we measured perceived social support and the quality of couples’ relationships concurrently with PTSD symptoms, it was impossible to determine the extent to which the emotional functioning of a person influenced their perception of relationships and support, potentially hindering their acceptance of help from loved ones. Finally, the study was conducted during various stages of the COVID-19 pandemic, which was a time of an increase in mental problems and a decrease in environmental support among postpartum women. Nevertheless, there was no association between the timing of sampling in relation to the pandemic status (lockdown) in Israel and the ASD and PTSD symptom levels. In terms of support during childbirth, although there were restrictions on the number of companions during the pandemic lockdown periods, all women were permitted one companion.

Future studies should focus on the risk and protective factors associated with specific ASD to PTSD symptoms trajectories and specific interventions, considering the differential role perceived social and partner support may play in different symptoms. For example, to examine whether a woman with predominantly general trauma symptoms may gain from interventions designed to strengthen environmental support resources (51), there is a need for longer, longitudinal studies measuring both partners.

## Conclusions and clinical implications

In conclusion, ASD symptoms measured shortly after birth were significantly associated with general childbirth PTSD symptoms and the total score measured at 8–12 weeks postpartum, but not with birth-related symptoms when considering birth type and levels of birth experience. In addition, social support and the quality of a couple’s relationship were associated with general childbirth PTSD symptoms, but neither of them was associated with birth-related PTSD symptom levels or the PTSD total score. PTSD birth-related symptoms and the total score were associated with the childbirth experience, while PTSD birth-related symptoms were also associated with birth type. This further validates the childbirth PTSD two-factor model and establishes the clinical importance of differentiating between birth-related and general symptoms. Thus, we suggest that clinicians focus specifically on women presenting the more general PTSD symptoms, which should be treated in order to avoid longer-term consequences, while providing help and reassurance for women regarding their more birth-related PTSD symptoms that may not be associated with longer-term effects. In addition, the results point to possible directions for targeted interventions utilizing specific support resources for women with specific symptoms.

## Data availability statement

The raw data supporting the conclusions of this article will be made available by the authors, without undue reservation.

## Ethics statement

The studies involving humans were approved by Rabin medical center review board. The studies were conducted in accordance with the local legislation and institutional requirements. The participants provided their written informed consent to participate in this study.

## Author contributions

JH: Writing – review & editing, Writing – original draft, Supervision, Investigation, Formal Analysis, Conceptualization. MK-H: Writing – review & editing, Writing – original draft, Methodology, Formal Analysis, Conceptualization. AR: Writing – original draft, Investigation, Formal Analysis, Data curation. YP: Writing – review & editing, Writing – original draft, Supervision, Methodology.
